# Designing an Integrated Low-cost Electrospinning Device for Nanofibrous Scaffold Fabrication

**DOI:** 10.1016/j.ohx.2021.e00250

**Published:** 2021-12-03

**Authors:** Hamza Abu Owida, Bashar Al-haj Moh'd, Mohammad Al Takrouri

**Affiliations:** a Medical Engineering Department, Al-Ahliyya Amman University, Jordan; b Electrical Engineering Department, Al-Ahliyya Amman University, Jordan

**Keywords:** Electrospinning, High voltage power supply, Syringe pump, Infusion pump, Nanofibers

## Abstract

Electrospinning is a cost-effective technique for synthesizing polymeric fibers with nanometers diameters. Electrospun nanofibers act as ideal scaffolds for tissue engineering and drug delivery systems because they can mimic the functions of native extracellular matrices. However, it is difficult to gathering nanofibers with simple design and reasonable price device. This study presents a cost effective and safe electrospinning system with similar capabilities to standard electrospinning device. As standard current electrospinning system consists of three constructed parts, a hand-constructed electrical power supply to provide a voltage source direct current (DC), a low cost three-dimensional (3D) printed syringe pump and handmade collectors. The device components are entirely constructed off-the-shelf components, and structural elements are 3D printer. The electrospinning process was carried out using PLA materials. The general parameters in the production process are resolution of the spraying rate μL/min and the power supply provides electricity in kilovolt. The prototype is an affordable device; its cost is around 99.5% less than the laboratory commercial devices. The average diameters of the fibers were determined from SEM micrographs with the aid of Image J software, which shows that the electrospinning device successfully produces fibres on a nanometer scale. Henceforth, this project can help educational institutions to have such electrospinning system with ultra-low cost comparing with readymade systems in the market.

## Graphical abstract

**Figure:**
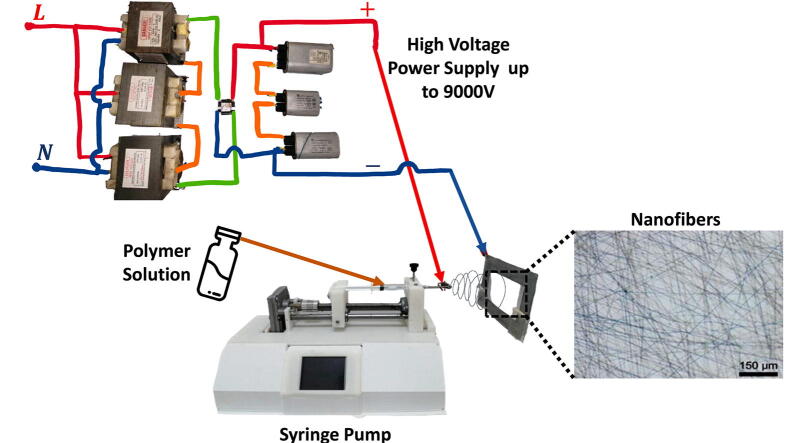

## Introduction

In 1959, Nobel Prize winner Richard Feymann put forward the revolutionary concept of designing molecular machines. Since then, the research community has delved further and further into the role that has played in the various aspects of research society what we now call nanotechnology. Basically, nanotechnology is the study and application of extremely small things ‘’nanoscale (1–100 nm)”. In recent decades enormous progress of nanotechnology has been reported and the recent development in nanotechnology has set very high standards in the biological and medical sciences [1], [2], [3]. Electrospinning has gained growing interest in the science community and industry since the late 20th century and is considered a critical scientific and commercial enterprise with global economic benefits. Electrospinning is a versatile and simple method to assemble continuous nanofibers [1]. The popularity of electrospinning is primarily due to the simplicity and versatility of the equipment and fabricate nanofibers with high surface area to volume ratio and large number of inter-/intra fibrous pores [4]. Standard electrospinning system comprises essentially of three parts: a High Voltage Power Supply (HVPS), syringe pump with steel needle, and a collector. Due to the potential difference between collector and needle on the syringe pump, an electrical field was created. There are two fundamental forces that influence of electrospun fibers fabrication, the surface tension force and the applied electric field. The electric field strength causes the solution to drop off the needle in a conical mode known as Taylor Cone. If electrical force overcomes the surface tension of the polymer solution, the charged droplet forms a jet from the Taylor Cone tip. It is pulled onto a thin fiber as the jet expands and undergoes a whipping action as it moves towards the collector. Due to the unsteadiness and repulsive forces produced within it, the jet splits into smaller fibers. During this process, the solvent gradually evaporates into the moving space between the needle and the collector, which ultimately leads to the collector’s continuous and thin fibers [4], [5], [6]. Electrospinning research currently has expanded dramatically since electrospinning produces nanofiber scaffolds for tissue engineering and wound healing applications because they can mimic the functions of native extracellular matrices, which helps the cells migration, proliferation and formation of tissues [1] (Table 1).

**Table 1:** Specifications table.

Hardware name	Electrospinning for Nanofibrous Scaffold Fabrication
Subject area	• Engineering and Material Science • Educational Tools and Open Source Alternatives to Existing Infrastructure
Hardware type	• Electrical engineering and computer science • Mechanical engineering and materials science
Open-Source License	MIT license
Cost of Hardware	202$
Source File Repository	https://doi.org/10.17605/OSF.IO/5YGWE

### Cost

Hence the development of electrospinning also involves spreading tissue engineering sector in Jordan. Given the current economic situation in Jordan; the lack of research resources in tissue engineering with the imperative to improve research to continue to succeed, in particular through electrospinning in tissue engineering research. However, in Jordan, there is no research associated to developing electrospinning systems. The current electrospinning system in this situation is compulsory and will play an important role in Jordan and has a huge impact on making a break in Jordan’s tissue engineering in both research and practical learning fields. Commercial electrospinning devices are somewhat expensive for educational and start-up research purposes. The price of a single electrospinning system, like in the research lab, may range between $17,000 to $ 60,000 USD. Prices differ according to brand and market inconstancy, hence each startup research lab should build up its own electrospinning device [7].

This project aims to develop low-cost Electrospinning Device for Nanofibrous Scaffold Fabrication (EDNSF) for tissue engineering application or for undergraduate practical learning by minimizing the cost of this electrospinning, the electrical system of the device is completely constructed and entirely built with disposable and off-the-shelf parts, and structural elements are fabricated using a consumer- grade three-dimensional (3D) printer. The total cost of the EDNSF is only $202.0 (see Bill of Materials) and is reduced significantly by the fact of repurposing secondhand microwave oven and using their step-up transformers and some other components available inside it. The other parts of the system such as syringe pump can be 3D printed using Acrylonitrile Butadiene Styrene (ABS) or polylactic acid (PLA) fiber.

### System requirements

**Fig. 1:**
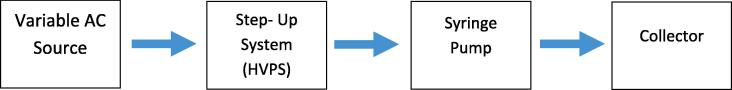
Electrospinning system block diagram.

A standard electrospinning device contains three main parts; HVPS; a syringe pump and a collector as shown in block diagram Fig. 1 [1]. Usually, in electrospinning process to produce nanofibers the applied voltage should be varied from 5 to 50 kV with optimized flow rate, volume and safety concerns [4]. For educational and initial research purposes, commercial electrospinning equipment is very costly. Prices depend on brand and consumer inconsistency, consequently every research laboratory start-up should build its own spinning system. To minimize electrospinning system costs, some low-cost electrospinning device studies have been developed.

Earlier, a low-cost electrospinning system with acrylic safety cabinet was designed and constructed at Universidad Autónoma de Baja California, Mexico, to provide undergraduate practical learning in the bioengineering field. To fabricate a poly (vinyl alcohol) nanofibrous scaffolds a commercial single syringe pump (WPI) SP120PZ) to deliver 0.5 ml with 0.2 ml/h of flow rate and commercial power (Spellman's Bertan Brand High Voltage Module, model: 605C − 200P) with 13 kV were used [7].

On the basis of electrospinning technique, Chanthakulchan et al. (2015) developed patterned nanofibrous scaffolds. In the proposed device, there are three main units: collector unit, sprayer nozzle unit, and a controller unit with commercial high voltage power supply. On the collector platform is a plate of non-conductive acrylic and the nozzle is a glass-syringe attached to a metallic needle. The control unit is made up of a control program, motion control board, digital board, local controller, and motor drivers. Two perpendicularly positioned ball screws guide the collector platform's movement, which is powered by two stepping motors [8].

Revia et al. 2019 developed and validated the use of mobile electrospinning systems to produce skin-direct nanofibers for hair stiffening cosmetic therapy. Two commercial HV DC-DC converters, one was providing a positive voltage (Q101-5, XP Power, Singapore) which are connected to ground and another providing a negative voltage (Q101N-5, XP Power, Singapore) with output voltage between 1 and 10 kV [9]. In this study the syringe pump was designed to deliver 1 ml of poly (vinyl alcohol) with 1 ml/h of injection rate which is comprised a stepper motor (QSH4218-35-10-027, Trinamic Motion Control, Hamburg, Germany) and structural part made with a 3D printer. For safety issues the mobile electrospinner device avoids any vital hazard by isolating the HV circuit from all mains-powered devices.

Another study built up a low-cost and HVPS by using a Zero Cross Voltage Switching driver circuit and a fly back transformer to provide 17 kV for the electrospinning method and fabricated a polycaprolactone nanofibers mesh. The commercial syringe pump (Chemyx Fusion 100) to deliver 2 ml with 1 ml/h of flow rate [10].

Using the electrospinning technique, Domínguez et al. (2021) designed, assembled, and validated a 3D printable prototype to obtain fibers. The prototype's particular configuration consisted of regulating the process conditions, according to the manufacturer with commercial high voltage power supply (B0788VTP9S). In the first module, injection rate and rotational speed of the collector can be controlled to produce fibers. Two modules control the collector's motion along the “y” axis, while the third controls the distance between the nozzle and the collector when it's in use. Syringe pump and collector are controlled by Arduino, and a 3D printed nozzle completes the device. On the other hand, the plunger-pressing mechanism is driven by an electric motor that rotates the threaded rod. There are three parts to the Collector: DC motor, collector holding device, and container with a lid [11].

In the light of the above studies, none of them have an integrated low-cost and effective electrospinning device that is not entirely developed low cost; power supply, syringe pump, and collector. The current electrospinning device components are entirely constructed with off-the-shelf and low cost components, and structural elements are 3D printer.

Herein, the chief components of current design include DC HVPS, a syringe pump driven by a stepper motor and collectors with plastic enclosure. The power supply has a simple main single switch with step-up transformer to produce high AC voltage, then rectifier circuit with high voltage capacitor for smoothing the signal to have pure DC voltage.

### System performance and safety

A HVPS with 9000 V has been built. Measuring such value is impossible with regular digital multimeters (DMM). In order to set forth a simple, precise, and easy to use system, the HVPS was provided with embedded voltage reading circuit with LCD. The voltage reading was accurate within 1%. The result was compared with a reading using DMM when the voltage was below 2000 V (below the maximum reading of DMM used). The syringe pump was comprised of a 12 VDC power supply, Arduino Nano, Nextion touch screen LCD, a stepper motor, the motor driver, and structural components fabricated with a 3D printer. The touch screen is relatively expensive comparing to the whole system cost. But, in fact, it makes the interface of the syringe pump system easier for the user. Controller and driver are assembled on PCB, the PCB is also containing wire-to-PCB connectors to easy assemble and disassemble the pump. Simple rectangular metal collectors have been reconstructed to be electrode – ground collector to receive fiber product. Since electrospinning process was carried out under high voltage power source that pose concerns about safety issues on account. The current electrospinning device avoids such hazards and guarantee working under safe operating conditions by isolating the high voltage circuit by acrylic safety cabinet. In addition, for safety issues the power supply box was built using wooden boards coated with insulation layer and implemented with Safety Switch which can cut off the main power instantly. Herein, the system provides a delayed time up to 3 min to check all the set up steps are ready before starting the spinning process. To reduce the risk of static shock and harmful solvent exposed for users, appropriate engineering regulations and safety and protective tools (lab coat, facemask, goggles, and insulator gloves) should be in spot for user safety. Before processing the equipment, users should ensure adequate electrical discharge at the spinneret tip. Wearing appropriate footwear with an insulating sole would indeed help to reduce static discharge.

## Hardware description

The EDNSF system consists of three main parts: (1) HVPS, (2) Syringe Pump, and (3) Collector. The proposed HVPS can provide DC signal up to 9000 V with embedded measurement system for the output voltage. The syringe pump has been completely designed using 3D printing. The pump is supported with touch screen, the touch screen can make any future development more flexible and easily modified as needed by reprogramming the Arduino controller and the touch screen. The collector should be a good electric conductor, such as aluminum; rectangle aluminum plates, (10 cm long, 4 cm wide, 0.5 cm thick) were made. In addition, a collector holder base was designed to support the collector and allow the distance between the collector and the needle tip of the syringe to be changed*.* All electronic circuits are designed as PCB’s. there are two electronic circuits in the proposed system: (1) HVPS measurement system, and (2) syringe pump controller. Design files can be found in the repository https://doi.org/10.17605/OSF.IO/5YGWE.

### High voltage power supply (HVPS)

**Fig. 2:**
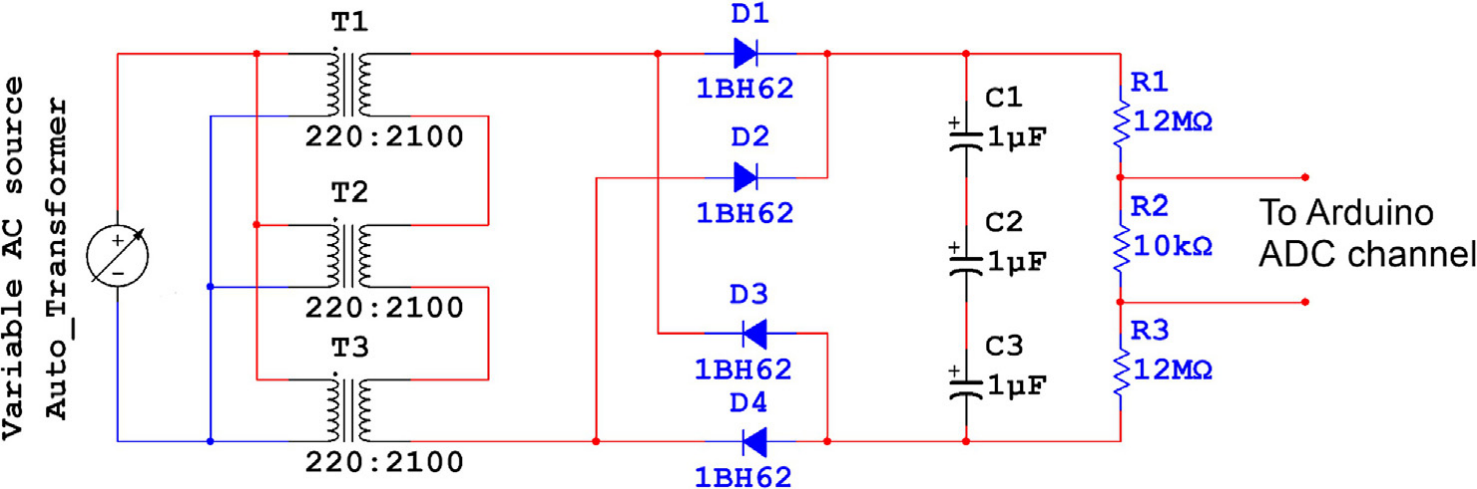
HVPS Schematic.

**Fig. 3:**
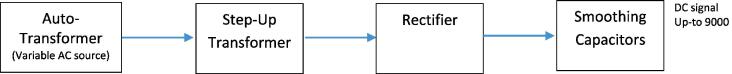
HVPS block diagram.

The HVPS has been designed and constructed with low cost with all the necessary specifications. The circuit diagram is shown in Fig. 2. The step-up transformer of ration (220:6300) is the core of this power supply. To avoid high-cost customized transformer; three transformers of the ratio (220:2100) with series connection have been used. The choice of these transformers is since they are available inside every microwave oven. Repurposed secondhand microwave ovens are available for less than 30$, which can help to build the required power supply. The secondary coils of the three transformers are connected in series in the same winding direction as shown in Fig. 2 (dot in the upper side of each transformer), while the primary coils are connected on parallel. In this case the output will be equal to the summation of the three voltages. After getting 6300 V of alternating current (AC), full wave rectifier is the next stage. The diodes in this case must have high voltage breakdown voltage, these diodes are relatively much expensive comparing with the commonly used diode such as 1 N4007, which has 1000 V breakdown voltage. Fortunately, the diodes are also available in the microwave ovens, and they have been used to complete building this power supply without any extra cost. Moreover, the capacitors for smoothing the rectified signal in the next stage of the circuit diagram are also taken from the same repurposed microwave ovens. Prior to the proposed power supply, an autotransformer as shown in Fig. 2 is being used to have variable AC as an input, and the output is variable high voltage DC signal up to 9000 V. The overall block diagram is shown in Fig. 3.

Where 8000 V is required, the series connection could provide up to 6300 V (RMS). Due to the rectification the capacitors will connect the peak of each half cycle with straight line to have very low ripple. So, the power supply can provide up to 9000 V DC signal, where the relation of the peak value and the RMS value is shown in equation (1). Using equation (1) the peak value will be equal to almost 9000 V.

(1) $$V_{\mathrm{peak}}=V_{\mathrm{RMS}}\times \sqrt{2}$$

**Fig. 4:**
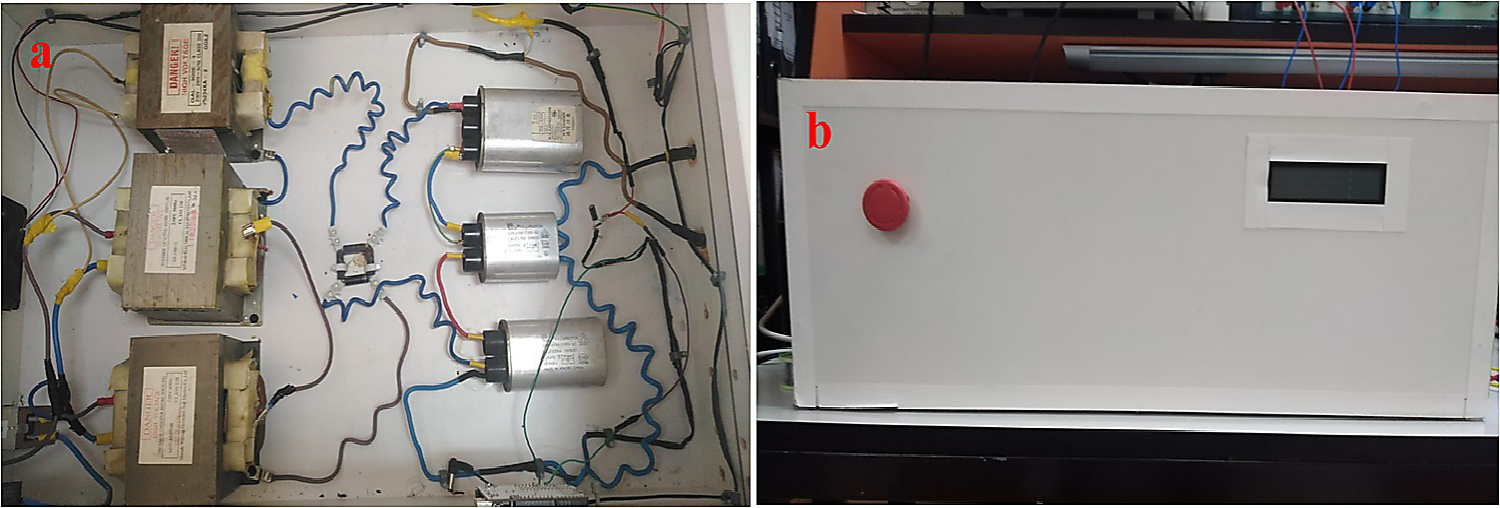
(a) HVPS circuit, (b) The front view of HVPS.

At the output stage, three capacitors are connected in series. Since each capacitor has 3000 V of breakdown voltage. Connecting three capacitors in series will make the breakdown voltage of 9000 V but this will reduce the equivalent capacitance. In our case this is not an issue, as the load is relatively small compared with the equivalent capacitance connection. Finally, to measure high voltage such 8000 V or 9000 V in a simple way, three resistors are connected in series at the output stage 12MΩ, 10kΩ, and 12MΩ respectively. The voltage across the 10kΩ (the resistor in the middle of the series connection) is being measured. Using the voltage divider, we can know that the output voltage is almost equal to 1/2400 of the output voltage across the two ends of the series connection, e.g., if the output voltage is 9000 V, then the voltage across 10kΩ is 3.75 V which is the maximum possible value in the proposed system. This range of voltage can be easily measured by ADC using “Arduino” board. In Arduino, the ADC module has 10-bits resulting 1024 level. Hence, each level represents 5/1024 which equal to 4.88 mV/level. In the Arduino algorithm, the ADC reading has been converted to the final output voltage using the formula (2) The results are shown on 2x16 LCD display as appeared on Fig. 4 (b).

(2) $$V_{\mathrm{out}}=4.88\times 10^{-3}\times {\mathrm{ADC}}_{\mathrm{reading}}\times 2400$$

Due to the resistance tolerance, high error in the reading is expected with the high voltage. Therefore, resistors with 1% tolerance are used. This makes the error due to the resistor tolerance not more than ± 50 V if the output is 5000 V (as an example).

**Fig. 5:**
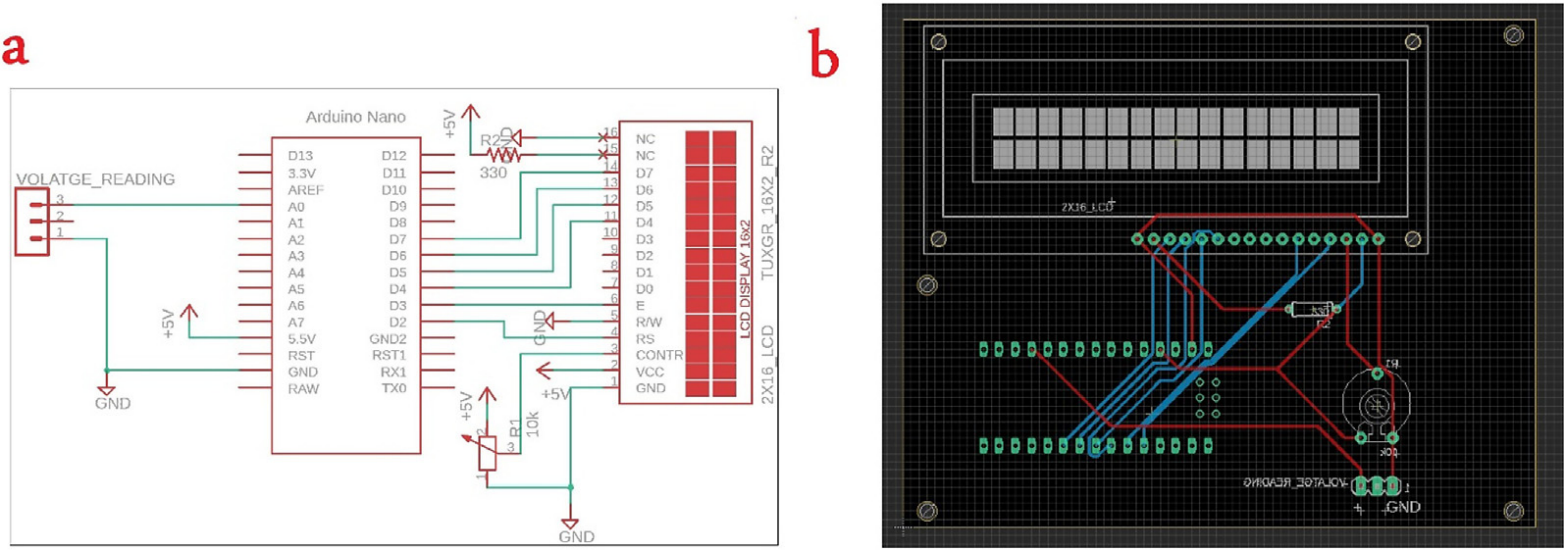
Voltage reading circuit, (a) circuit schematic, (b) PCB layout.

The front view and the top view of the device are shown in Fig. 4. The system is manually connected and enclosed by wooden case. Voltage reading circuit schematic and PCB layout are shown in Fig. 5. The circuit is simply including an Arduino Nano, 2x16 LCD, and connector. The voltage reading connector on the PCB should be connected across the 10kΩ resistor as shown in the series connection in Fig. 5. Because PCB include LCD for user interface and for safety purpose, we have avoided to put the series connection of the resistors on the PCB to avoid high voltage pass to the user. It is manually connected and fitted on the base of the wooden box.

### Syringe pump

Infusion pumps usually costs $800 or more, current project’s pump has been fabricated by 3D printer and cost less than $75. The final shape is shown in Fig. 10. The hardware includes Arduino-Nano board, stepper motor, stepper motor driver, gear box, touchscreen for to control and monitor the process, and basic hardware components. The designed pump will provide a smooth delivery for the liquid comparable with most popular IV standard set. The hardware of the syringe pump can be described by dividing them into three parts: printed parts, non-printed parts, and touch screen and user interface.

#### Printed parts

Nowadays the prices of 3D printers are not limited only to prototyping but also to produce the end product, due to the fact that they produce (with the right materials and design) a reliable robust part without many hardware requirements other than the 3D printer itself and the printing material. In addition, 3D printers prices are decreasing sharply in every part of the world [12]. These are the main reasons which made many institutions such as universities to use them in their laboratories and research and development departments. In this design project, the syringe pump was tailored for the electrospinning by using a 3D CAD designer. The used 3D designer software is from Autodesk. Autodesk Fusion 360 is a software allows the user to build any project from the scratch or import and configure any already made CAD design. The printed parts can be created by using PLA or ABS. ABS is known for being more rigid and tolerates higher working temperature without losing its form. On the other hand, PLA tends to droop and deform as the temperature approaches the glass transmission of $60^{o}C$ [13]. However, for this application PLA has enough durability and the usual working environment for electrospinning is around the room temperature.

**Fig. 6:**
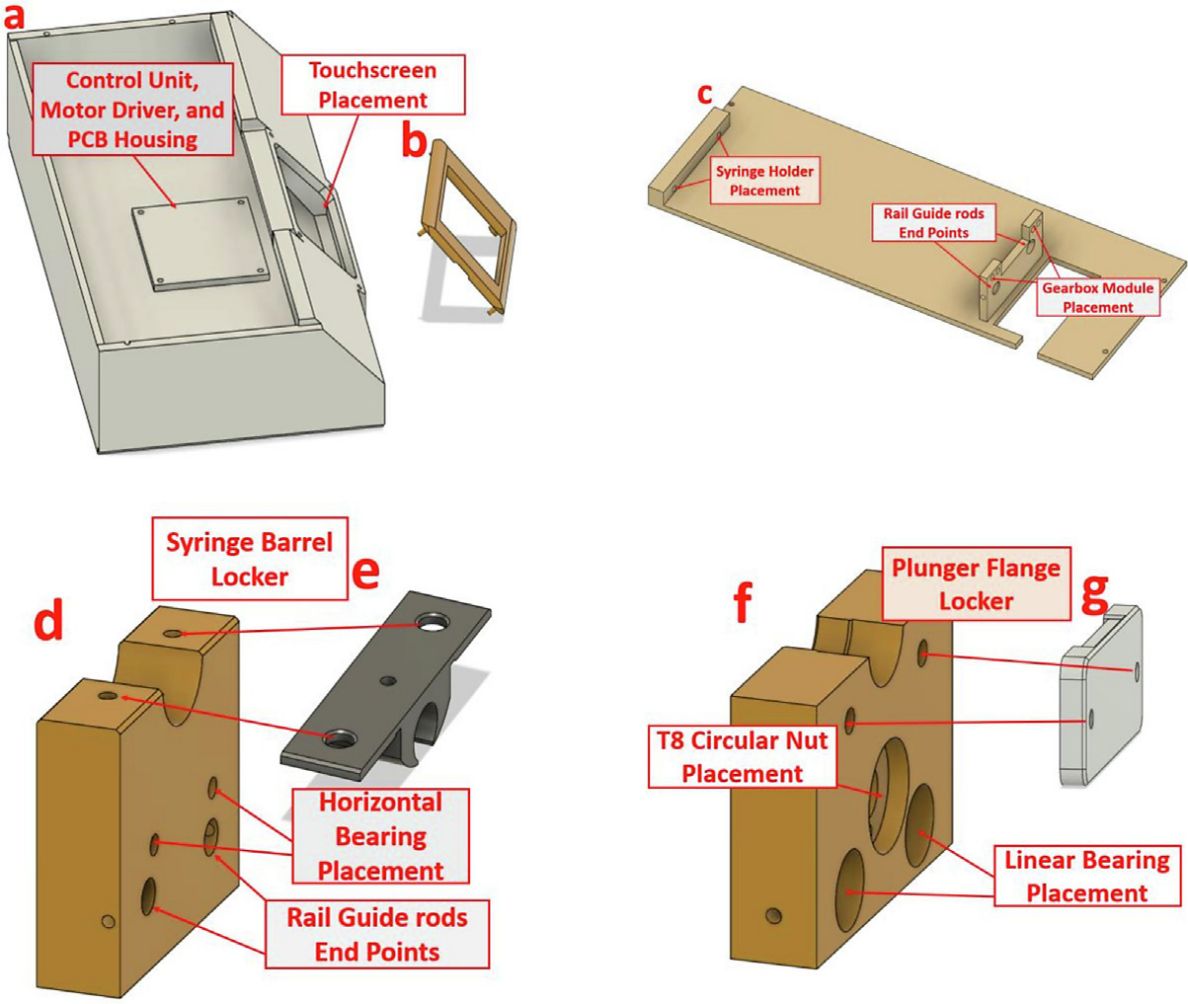
3D CAD design of syringe pump highlighting several features, (a,b) the main case and touchscreen front frame, (c) top cover, (d,e) syringe barrel holder and fixer, (f,g) middle free moving part and plunger flange locker.

The full design is made up of several smaller parts rather a solid block. This approach was followed in order to have a customized dimension and to make editing easy for the builder to fit their specification. The design consists of 4 main parts. The main case seen in Fig. 6 (a) will provide a place to mount the touchscreen, to fix the electronics, and to house different types of stepper motors. More detailed information of the used electronics, stepper motor, and gears will be provided in section 2.2.2. The small piece shown in (b) can be considered a part of the main case and it is used as a front cover for the touchscreen.

On the top of the main case, we have the second part which has the structure to fix and tighten all the essential parts of the syringe pump such as the gear box and the syringe holder. Fig. 6 (c), shows a part the features of the top cover and the fabricated M3 holes.

The syringe barrel is mounted onto the top of the designed piece shown in Fig. 6 (d), this piece is configured to be able to use any barrel size. However, the part in Fig. 6 (e) was designed specifically to house a 3 ml syringe. Additionally, a KFL08 horizontal bearing is fixed on this part as it will show in 2.2.2.

The final and fourth part of this CAD 3D design which links the shaft of the gears with syringe barrel shown in Fig. 6 (f), which is a free moving component where it transfers the force from the shaft to plunger of the syringe. This part is guided by two linear rods held in their places by linear bearings, these components are common for 3d printers and easily obtained. The plunger flange of the syringe is held on the component by using an additional CAD part and tighten using M3 bolts and nuts as displayed in Figure (g). Finally, it is moved freely by a leadscrew and a circular nut as known as T8 rod lead screw. The lead screw is about 130 mm in length. Fig. 6 shows the main four described 3d printed part in consequential order.

**Fig. 7:**
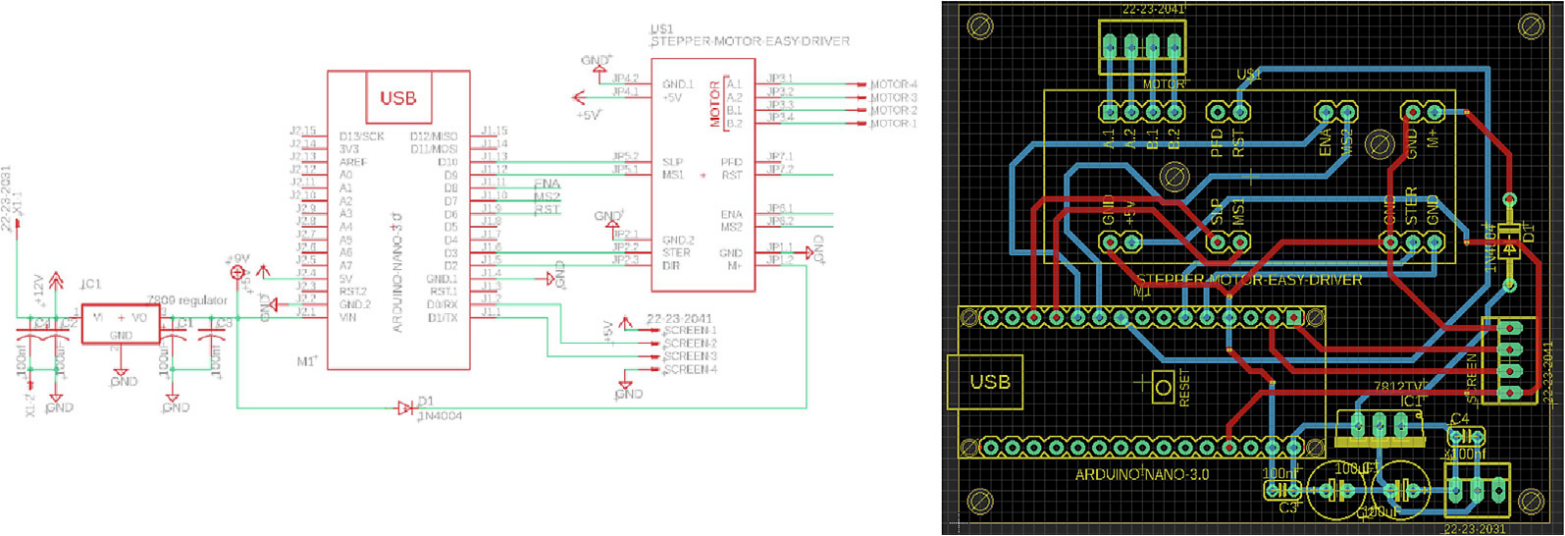
Syringe pump controller circuit, (a) schematic connection, (b) PCB layout.

In addition to the 3D printed parts, an optional component can be placed in this category which is the PCB board. The user has the choice to print the PCB and solder the electronics or to wire the components directly. The designed PCB for the syringe pump has the purpose to connect the controller (Arduino Nano), a 12 V / 9 V regulator, a motor driver, and provide kk connectors to hook up the touchscreen and the stepper motor. More information on the electronics and the stepper motor are mentioned in the section 2.2.2. Fig. 7 show the schematic connection of the components and the PCB layout.

#### Non-printed parts

The nonprinted parts consist of the electronics, gears, stepper motor, and standard hard such as M3 screws and nuts which will be described in detail. Arduino uno was chosen to be the control unit of the system due to its wide availability and its sufficient I/O pins to control the stepper motor via the driver and to operate the touchscreen.

A3967 is used to drive the syringe pump, where this motor driver is the most common for all stepper motors as it enables the builder to easily connect any stepper motor at hand for the system. Due to the fact that the syringe pump does not require high torque, an off the shelf 35 mm stepper motor is picked. However, any other stepper motor model (e.g., NEMA motors) can be used in this system and an adapter can be easily designed in order for the motor to fit with the gears as will be discussed.

The syringe pump performance is monitored and controlled via a touchscreen where it will be mounted on the front of the structure. Nextion Display 2.8″ was selected for this task as it is compatible with Arduino uno and it is easily configured. However, this component is not essential for the system, and it can be replaced by simple push buttons. In this paper the touchscreen usage is detailed in 2.2.3. Finally, the electronics and the 35 mm stepper motor are powered by a standard switch mode 12 V power supply with capability of providing with no less than 1A which is easily available. The 12 V power source is regulated to 9 V to match the rated voltage of the used stepper motor. However, the regulator is not necessary for other types of motors. The connection of the previously mentioned parts is shown in Fig. 7, where the user can use simple jumper to skip the regulator.

The touch screen display and the stepper motor cannot be seen in Fig. 7 due to the fact that the design was made on PCB eagle which is software used to produce the final PCB files. However, each of these components follow simple wiring and their corresponding connection to the system can be seen in the schematic. Fig. 7 shows the tracing of the copper on the PCB board, where kk connectors are installed to provide easy wiring for the touch screen and the stepper motor.

**Fig. 8:**
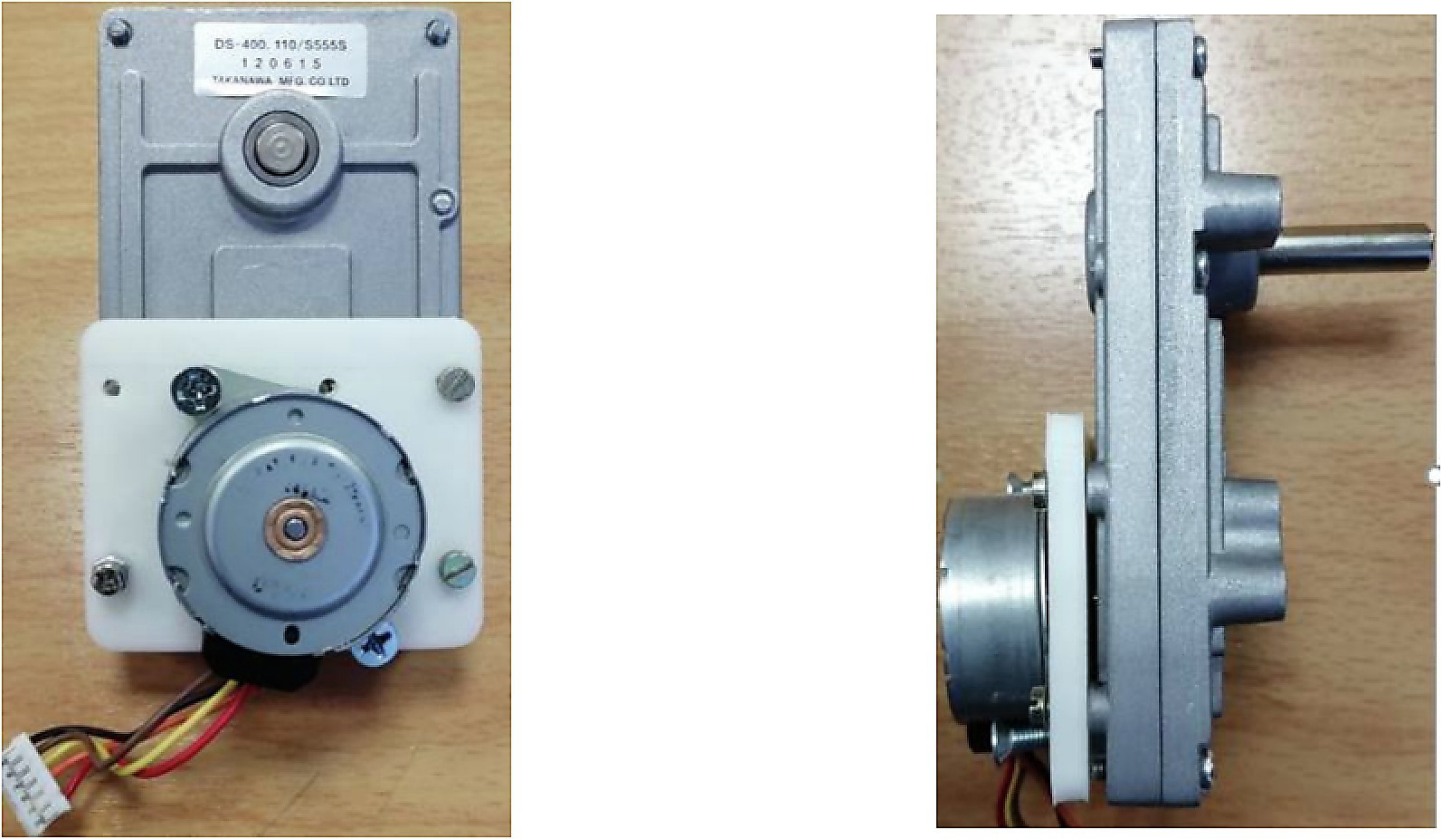
35 mm stepper motor adapted to the salvaged gears.

Other hardware non printed parts are standard M3 bolts, M3 nuts, T8 leadscrew and its circular nut, linear bearings, circular bearings, and rail guide 8 mm shaft. Amounts, prices, and a link for provider are detailed in section 4) Bill of Materials. Finally, salvaged gearbox module from a robust and well available geared motor was adapted for our 35 mm stepper motor. The small stepper motor was connected to the gearbox by using a simple CAD design, the mounted stepper motor on the gearbox from front and side view is shown in Fig. 8 below.

**Fig. 9:**
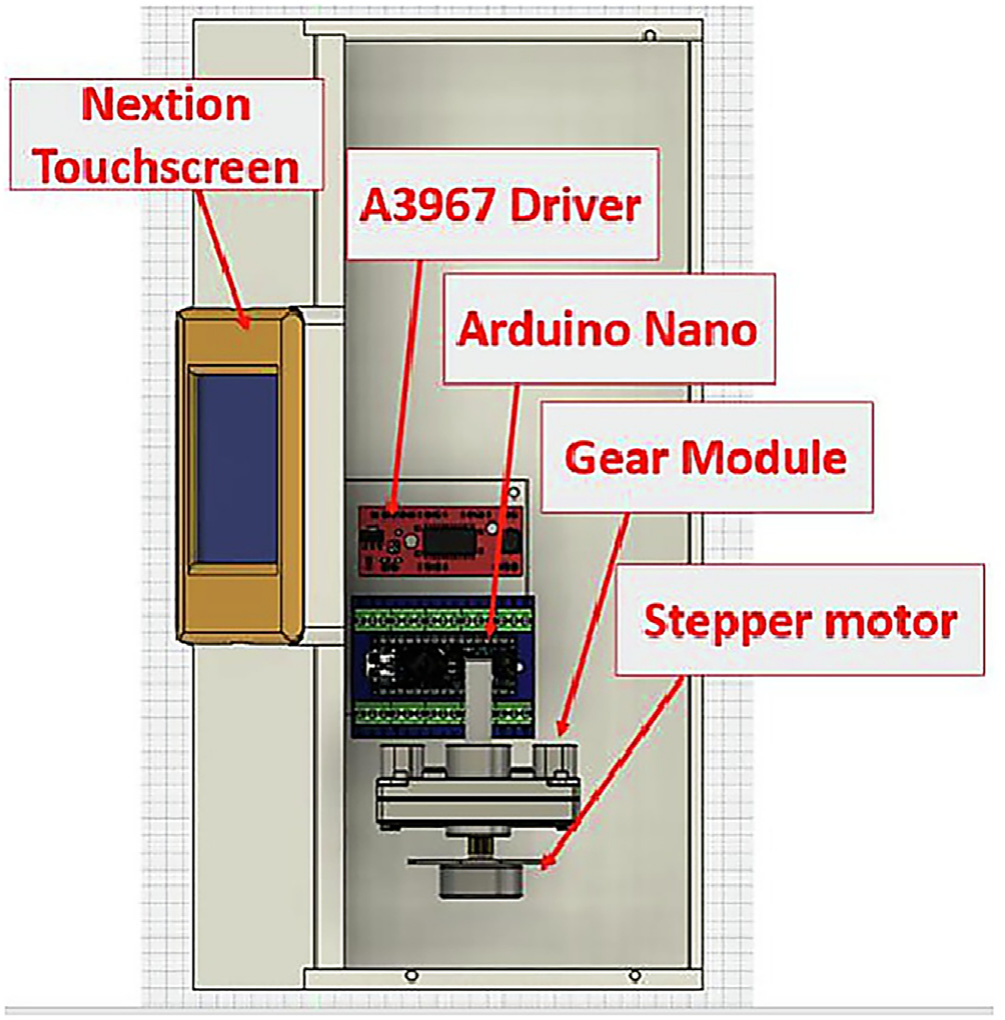
Top view of the electronics, stepper motor, and the gear module.

**Fig. 10:**
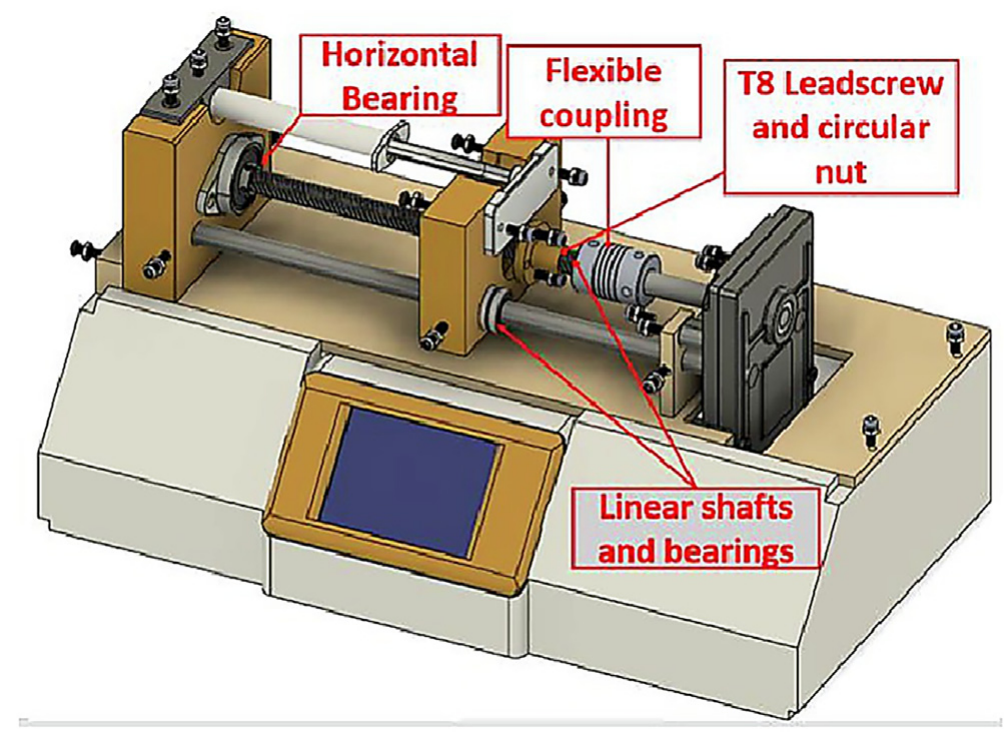
syringe pump overall appearance in 3D.

The top view of the described components is shown in Fig. 9, where it shows the touchscreen, the motor driver, the controller, and the gear module. The 3D design of the printed parts was made to take into consideration if the user has chosen a different approach regarding the stepper motor, for example if the builder has picked Nema motor instead of the 35 mm stepper motor used in this design it would a have sufficient area inside the main case to house it. The final appearance of the syringe pump highlighting the used hardware parts is shown in Fig. 10.

#### Touch screen and user interface

**Fig. 11:**
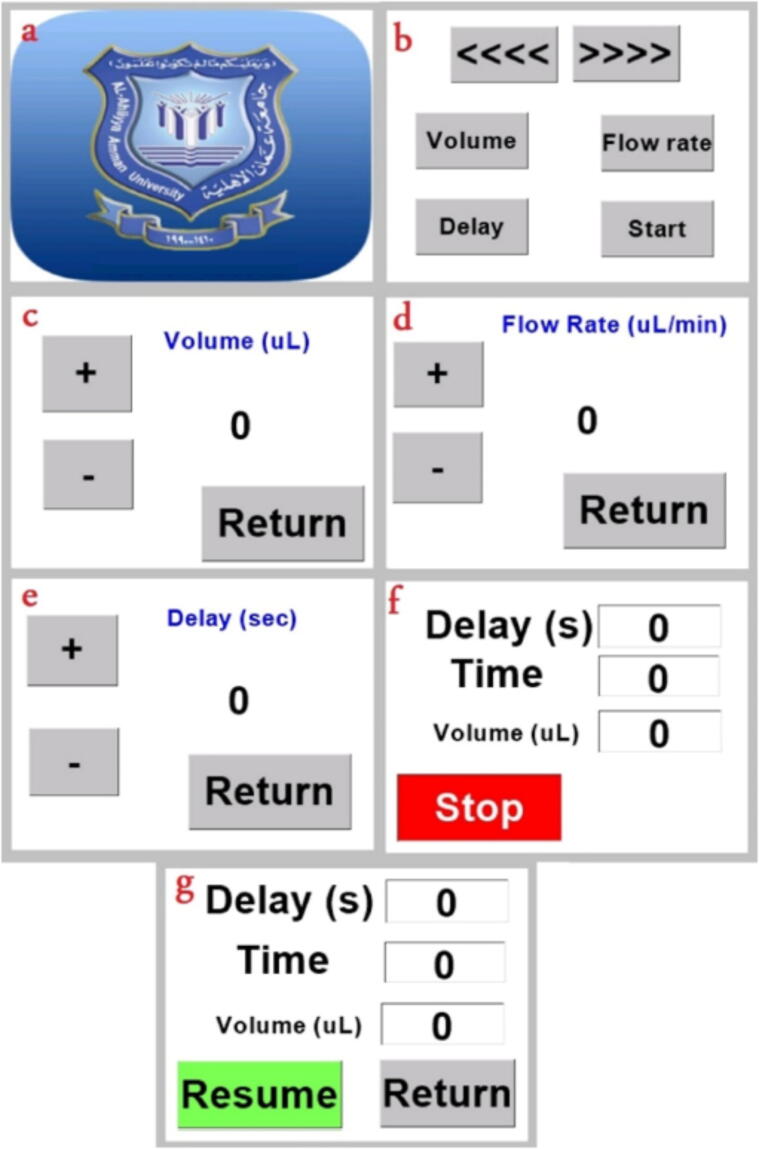
Touch screen pages (a) Introduction page, (b) main page, (c) Volume setting page, (d) Flow rate setting page, (e) Delay setting page, (f) Monitoring page, (g) Monitoring page when operation paused.

As previously mentioned, the syringe pump operation is monitored and controller using a touchscreen. Since this build used 3 ml barrel size, some calculations were based on it, more information on the calculations are mentioned in 5.2 (Build instructions). However, this design used Nextion 2.4″, which is easily programmed and controlled by Arduino Nano, the codes are available in 3 (Design files)3. The touch screen interface pages are shown in alphabetical order in Fig. 11, where each panel is described as follows:

Fig. 11 (a) represents an optional page which is only shown once the system is powered, it will take five seconds to go to the next page (the main page).

The main page in (b), where on the top of the screen gives the operator the ability to manually move the syringe pump forward or backward, i.e., to inject the polymer into the needle or withhold it. This home page links to all configurable settings and the button for commence the polymer pumping.

“Volume” option is shown on the top left of the main page where it takes the user to the panel (c) in Fig. 11. Volume settings gives the ability to set the volume of the polymer to be inject into the needle, the user can increase or decrease by touching the [+] or [–] signs and go back to the home page by pressing “Return” button.

The flow rate of which the polymer is injected is controlled by taping “flow rate”. The flow rate is adjusted in [μL/min] shown in Page (d), which in its role changes the speed of the stepper motor.

The final adjustable parameter in the home page (b) is the delay, this option was designed for safety and to give the operator preparation time to check other components of system (e.g., the collector) are in their appropriate placements. The delay configurations in page (e) are adjusted in the same manner to the other settings.

Finally, the user can begin the injection process with the button “Start” and the interface will change to the panel (f) where three quantities are monitored; a countdown of the remaining delay, the elapsed time, and the amount of the polymer to be delivered in the barrel. The “stop” option in page (f) gives the preference to the user to pause the process and resume it later or to return to the main page as demonstrated in (g).

### Collector

A rectangular portable collector made up of a conductive material frame with conductive wires attached to the negative electrode was used to produce nanofibers meshes with low line density. General set-up and validation of electrospinning system is used to fabricate nanofibers mesh. The established protocol in the lab was adapted to fabricate nanofibers mesh [14]. 2% poly-l,dlactic acid (PLA) solution was prepared by dissolving PLA granules in a mixture of chloroform and N, NDimethylformamide solvents in a (7:3) ratio to produce nanofibers. The resulting 0.2 ml PLA solution was delivered at a 25 µL/minute flow rate by our lab designed syringe pump through an 18G needle attached to the positive electrode. The distance between the positive and the collector (the negative electrode) was fixed to 15 cm. The needle and the collector were connected to our lab designed power supply charged at ± 8 kV. The collector should have a perfect electric conductivity, such as aluminum; rectangle aluminum plates, (10 cm long, 4 cm wide, 0.5 cm thick) were made. Besides, a collector holder base was made to provide collector support and manage the distance between the collector and the syringe's needle tip to be changed.

## Design files

Design Files Summary

**Table:** 

Design file name	File type	Open source license	Location of the file
Syringe pump controller PCB (Fig. 7)	Eagle CAD schematic, PCB layout, and Gerber files in one zip file (Extended RS-274xx)	MIT license	https://doi.org/10.17605/OSF.IO/5YGWE
Syringe pump code for the Arduino Nano on the PCB (Fig. 7)	Arduino IDE (.ino)	MIT license	https://doi.org/10.17605/OSF.IO/5YGWE
Nextion touch screen software	Nextion Editor (.HMI)	MIT license	https://doi.org/10.17605/OSF.IO/5YGWE
Syringe pump “Main_case” (Fig. 6(a))	CAD file & STL file	MIT license	https://doi.org/10.17605/OSF.IO/5YGWE
Syringe pump “Front_cover_for_touchscreen” (Fig. 6(b))	CAD file & STL file	MIT license	https://doi.org/10.17605/OSF.IO/5YGWE
Syringe pump “Top cover” (Fig. 6(c))	CAD file & STL file	MIT license	https://doi.org/10.17605/OSF.IO/5YGWE
Syringe pump “Plunger_flanger_holder_p1” (Fig. 6(d))	CAD file & STL file	MIT license	https://doi.org/10.17605/OSF.IO/5YGWE
Syringe pump “Plunger_flanger_holder_p2” (Fig. 6(e))	CAD file & STL file	MIT license	https://doi.org/10.17605/OSF.IO/5YGWE
Syringe pump “Syringe_holder_p1” (Fig. 6(f))	CAD file & STL file	MIT license	https://doi.org/10.17605/OSF.IO/5YGWE
Syringe pump “Syringe_holder_p2” (Fig. 6(g))	CAD file & STL file	MIT license	https://doi.org/10.17605/OSF.IO/5YGWE
Syringe pump “35 mm adapter” (Fig. 8)	CAD file & STL file	MIT license	https://doi.org/10.17605/OSF.IO/5YGWE
Voltage Reading PCB (Fig. 5)	Eagle CAD schematic, PCB layout, and Gerber files in one zip file (Extended RS-274xx)	MIT license	https://doi.org/10.17605/OSF.IO/5YGWE
Voltage Reading Arduino Source code	Arduino IDE (.ino)	MIT license	https://doi.org/10.17605/OSF.IO/5YGWE

Bill of Materials

**Table:** 

Designator	Component	Quantity	Cost per unit (USD)	Total cost (USD)	Source of materials	Material type
Nextion Display 2.4″ Touch screen-Syringe pump	NX3224T-28	1	21	21	AliExpress-Nextion_Display	TFT LCD
Flexible Coupling-Syringe pump	Coupling GR 8x8	1	5.52	5.52	AliExpress- Coupling GR Aluminum	Aluminum Alloy
Screw leader + Circle nut Syringe pump	T8 Leadscrew + Brass Nut	1	2.97	2.97	AliExpress- T8 Leadscrew	Screw made with steel Nut is made with Brass
Lead bearing-Syringe pump	KFL08 Bearing 8 mm	1	1.24	1.24	AliExpress- KFL08 Bearing	Stainless steel
Rail-guide 8 mm rod shaft Syringe pump	Kande Bearings 8 mm 200 mm	2	5.14	10.28	AliExpress- Kande Bearings 8 mm 200 mm	Steel (C45 or Gcr15)
Linear Bearing - Syringe Pump	LM8UU Linear Bushing 8 mm	1 lot (2 pieces)	0.92	0.92	AliExpress- LM8UU Linear Bushing 8 mm	Iron
M3x8mm screw bolts - Syringe Pump	M3x8mm Steel Screws Bolts Thread Nuts	18	0.06	1.08	AliExpress- M3x8mm Steel Screws Bolts	steel
M3x12mm screw bolts - Syringe Pump	M3x12mm Steel Screws Bolts Thread Nuts	10	0.04	0.40	AliExpress- M3x12mm Steel Screws Bolts	steel
M3 nuts - Syringe Pump	M3 Stainless Steel Hex Hexagon Nut	28	0.03	0.84	AliExpress- M3 Stainless Steel Hex Hexagon Nut	steel
Arduino Nano-Syringe pump	V3.0	1	14.95	14.95	Sparkfun- Arduin_nano_link	other
Stepper Motor	M35SP-7 T 35 mm stepper motor	1	1.29	1.29	AliExpress- 35 mm stepper motor	Steel – copper – iron - other
Stepper Motor driver	EasyDriver V4- A3967 microstepping driver	1	14.95	14.95	Sparkfun- Driver_Link	Other
Gauge Luer-Lock Needle- Syringe pump	Dispensing Needles Syringe Tip Needle	1	0.1	0.1	AliExpress- Dispensing Needles Syringe Tip Needle	Metal- plastic
Microwave oven 220:2100 transformer & 2100-volt capacitors- HVPS		3	10	30	Taken from secondhand microwave oven	Other
Arduino Uno for HVPSHVPS		1	7.91	7.91	AliExpress- Arduino_Uno_link	Other
LCD 2x16- HVPS Fig. 11-c)	1602 2x16 Big Characters 5 V 122*44 mm Dots Graphic Backlight LCD Display Module	1	9.51	9.51	AliExpress- LCD_link	Other
Inlet Module Plug Fuse Switch Male Power Socket (Fig. 11-a)	3 Pin IEC320 C14 Power Socket 10A 250 V	1	10.99	10.99	Amazon Inlet_Plug	
Safety Switch (Fig. 11- b)	ABB brand	1	10	10	Local retailers	
Banana socket (Fig. 11-d)		2	1	2	Local retailers	
Test leads “banana-to-crocodile” pair wire cable		1	7	7	Amazon banana-to-crocodile	
connectors cable wire FDD2-250		20	0.1	2	Local retailers	
Power supply 12 V/2A	For Syringe pump and voltage reading circuit	2	5	10	Local retailers	
PCB	Up to 10x10 cm	5	0.4	2.00	Upload (.zip) gerbers file at https://JLCPCB.com	FR4- 1.6 mm thickness
Wooden Box	64x40x26cm	1	15.0	15.0	University workshop	Wood 18 mm thickness
Acrylic cabinet	51x21x25	1	20.0	20.0	Local workshop	4 mm thickness of transparent acrylic

Simple electronic components such resistors, capacitors, diodes, connectors, and the 7809 regulator were taken from the labs or on-the-shelf. Details of parts values and numbers are provided in the schematics.

## Build instructions

A majority of this system are the HVPS, and the syringe pump. The main components of the HVPS are taken from the secondhand microwave ovens. The syringe pump parts are 3D printed. The remaining parts are very common and can be bought online or from electronics and hardware retailers. Follow the step-by-step procedure to rebuild such system.

## Build instructions for high voltage power supply

The HVPS is shown in Fig. 4. The outer box was built using wooden parts. The following steps describe specifically how to rebuild such system:

1. Buy three secondhand microwave ovens. From each oven take the following components from it:
• Step-up transformer as shown in Fig. 4 (a) Make sure that all transformers are between 800-to-900 W.
• The high voltage capacitor as shown in Fig. 4(a).
• The high voltage diodes as shown in Fig. 4(a)

You may need to buy a fourth diode to build a full wave rectifier, or alternatively you can just build a half wave rectifier as the required current is relatively small compared with the equivalent capacitance connection.

**Fig. 12:**
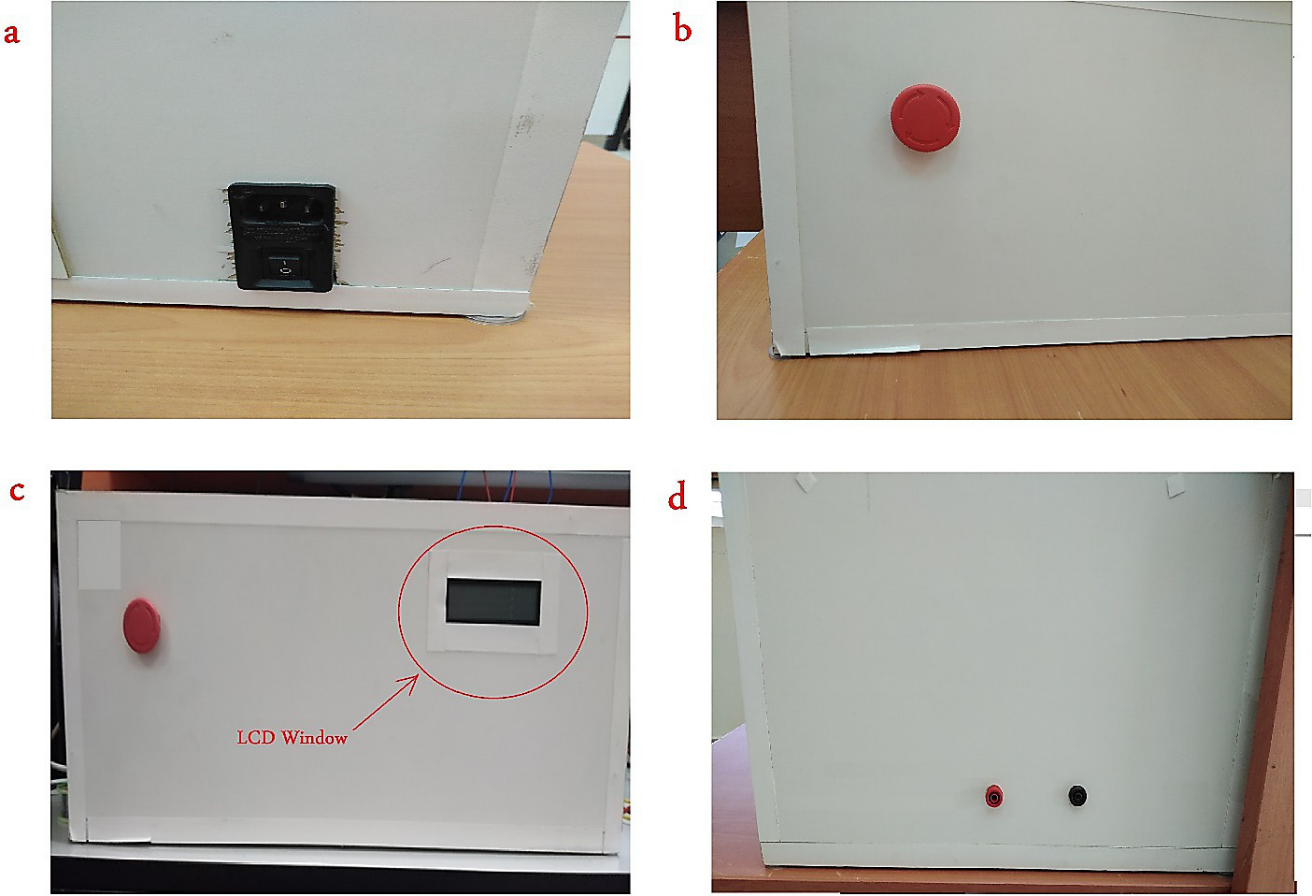
HVPS Wooden box (a) Inlet Module Plug Fuse Switch Male Power Socket, (b) Safety Switch, (c) LCD window, (d) Banana Sockets.

2. Build a wooden box of size 64x40x26cm and make it opened from the top as shown in Fig. 12. Leave three opened windows in this box for the following components:

(you can customize any of the dimensions in this part depending in the material and components used)

• Rectangular window from the left side to fit the “Inlet Module Plug Fuse Switch Male Power Socket”.
• Rectangular Window at the upper-right corner to fit the LCD display.
• A circle hole at the lower-left corner to fit the safety switch.
• Two circle holes of diameter 1 cm from the right side for the banana socket for the output voltage.

Note that: the autotransformer is a readymade device, and it is not a part of the wooden box. The output of the autotransformer will be connected to the “Inlet Module Plug”. See (Fig. 18 (a)).

3. Fit the “Inlet Module Plug” and the safety switch to the wooden box, then connect the circuit diagram shown in Fig. 4(a). Cut all the wires of the transformers The input of these transformers are the two pins as shown in Fig. 13(a) and the output can be taken from the chassis with pin at the other side as in Fig. 13(b).

**Fig. 13:**
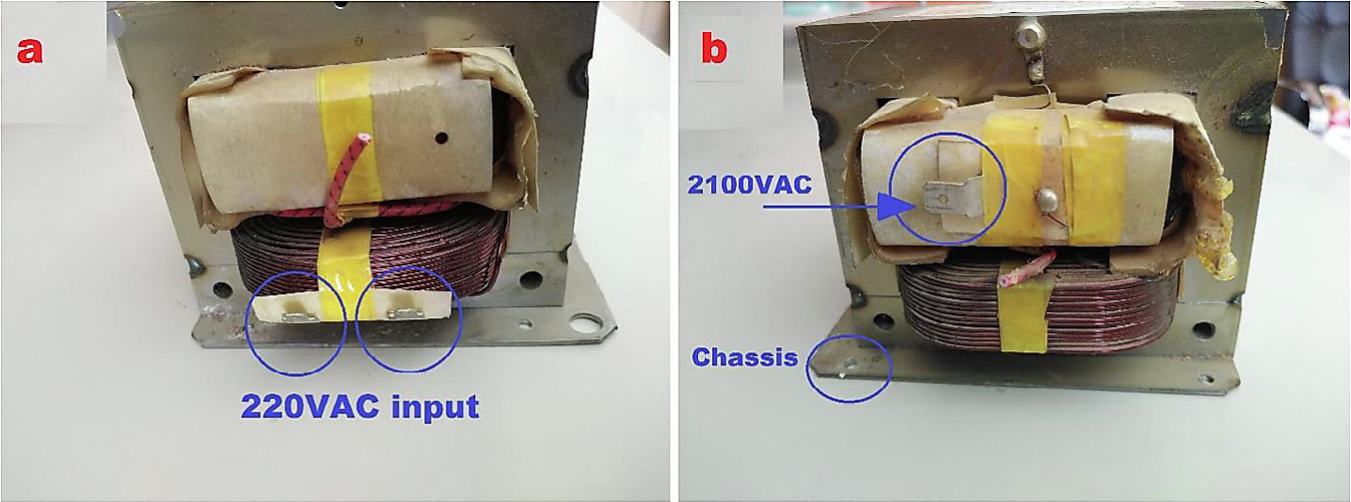
Step-up transformer (a) input pins (b) output pins.
4. Connect the series connection of the resistors manually to the end of the circuit (at the output stage) and take too long wire across the 10kΩ to be connected to the display board. For safety purpose, it is highly recommended to isolate every single component using shrinkable tubing and use the “connectors cable wire FDD2-250” for the connections between transformers and capacitors. Solder other unpluggable points. Note that, the 12MΩ resistors should have at least 2 W of rated power and tolerance no more than 1%, or alternatively you can connect series connection of smaller resistors in value and rated power (e.g. using 12 resistors of 1MΩ/0.25 W).
5. Print the PCB in Fig. 5 or alternatively, use strip board to connect it as in the schematic.
6. Connect the output across the 10kΩ to the display board throughout “wire to PCB connector”. Make sure to connect the positive side to “A0 pin” in Arduino and the negative side to the ground.
7. Upload the software “Vout” using Arduino IDE.

### Build for the syringe pump

1. Edit and make sure the CAD files fit your configurations as discussed in 2.2, where two considerations must be taken into account:
a. First, the assembler needs to adapt the design to fit his stepper motor of choice specifically for the top cover as the main the case can accommodate most stepper motor sizes.
b. Secondly, the total length from the gearbox to syringe barrel holder is about 180 mm. Hence, the design was made to use a 135 mm T8 Lead screw and 180 mm 8 mm rail guide shafts, the reader of this design paper must either edit the CAD design files to be compatible with his lead screw and shafts or cut them to match the design size.
2. After checking the considerations in the previous step, 3D print the CAD files listed in 3. The print was done by the use of PLA material and setting low infill parameter of about 15%. All the parts can be placed as flat on the 3D printer bed, accordingly they don’t require support structure.
3. If 200 mm rail guide rode is used and the CAD files and were not edited, cut the threaded rod to 180 mm and the same case for T8 screw which has to be cut to 135 mm.
4. Install the circuit nut inside the moving part piece and insert the linear bearings as indicated in Fig. 6 (f). The T8 circular nut needs to be inserted by using a hammer. On the other hand, the linear bearings can be fixed in their place by using M3 screws from the sides. Since the pieces are made with PLA the assembler can use any larger screw bolts without difficulty. After the linear bearings are installed correctly, insert the rail guide rode through the linear bearings.
5. For the part in Fig. 6 (d), install the horizontal bearing using M3 bolts and then tighten the T8 lead screw inside the bearing. Insert the two rail guide rods into their prospective holes and tighten them from the side in the same manner as in the previous step. Steps 4 and 5 are illustrated in sequential order in Fig. 14.

**Fig. 14:**
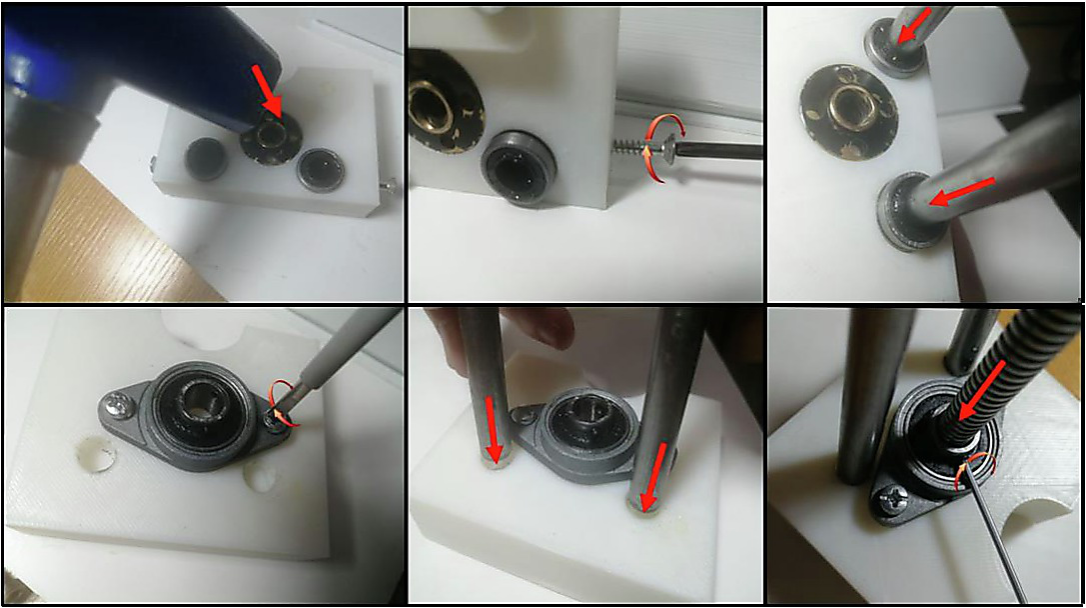
An Illustration of the assembly procedure for step 4 and 5.
6. Insert the rail guide rods in their holes on the top cover shown in Fig. 6 (c), and by using screws fix the barrel holder part on the other end. Pay attention that at this point, the rail guides and T8 lead screw should be in perfect parallel.
7. Insert the coupling inside the T8 lead screw and then fix the gearbox with the stepper motor in their place by using M4 bolts.
8. Tighten the flexible couple on the gearbox shaft and the leadscrew.
9. Last part for the mechanical assembly is to fix the syringe on the top of the free moving part and the barrel holder, by using the pieces in Fig. 6(e, g). The part (e) should be tightened on the top of part d and by using a M3 screw, it should apply suitable force to fix the syringe barrel in its place. The plunger flange on the other end is locked by using the part g.
10. The touch screen is easily assembled on the main case (Fig. 6(a)) by using the part b and M3 screws.
11. Place the electronics inside the main case or if the PCB is printed, fix it in its prospective place using M3 bolts. The steps 6 through 10 are shown in order in Fig. 15.

**Fig. 15:**
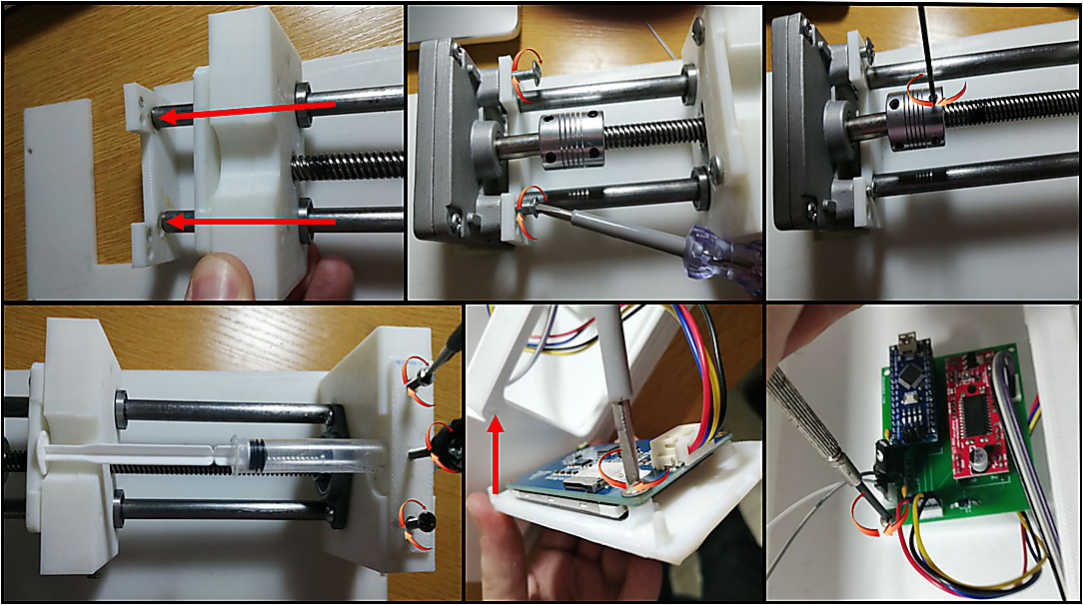
Build instructions for steps 6 to 10.
12. Finally, connect the controller, touchscreen, driver, stepper motor, and the power supply showed in the schematic of Fig. 7. If the provided PCB design was used, the user only has to connect the power supply, the four pins of the touch screen, and the four coil wires of the stepper motor to their kk connectors. The assembly should look as shown in Fig. 10 or as built in Fig. 16

**Fig. 16:**
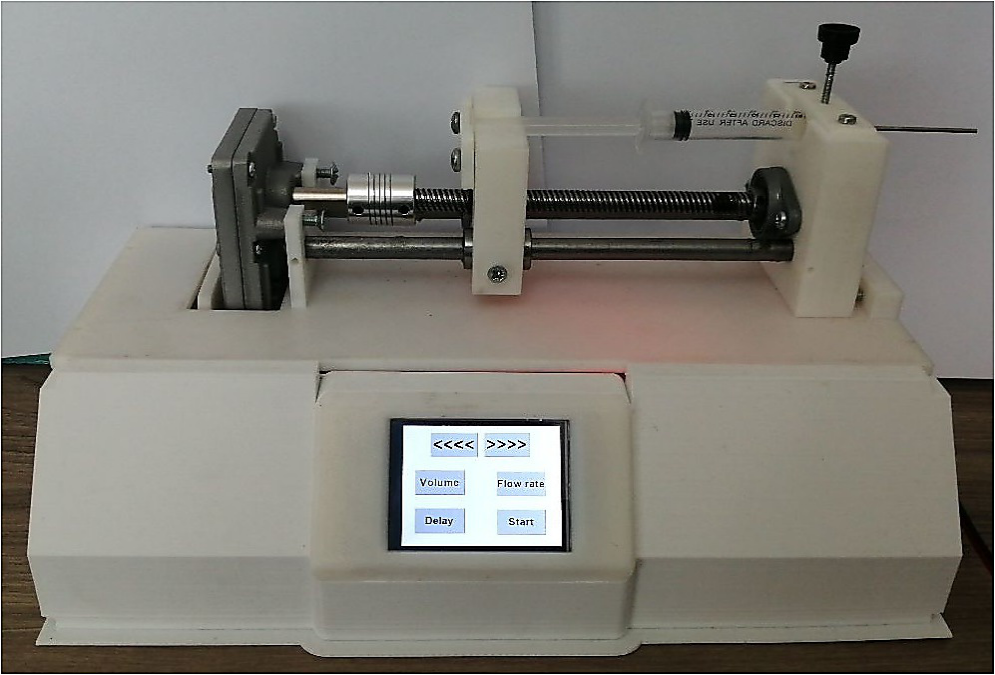
Final view of the assembled syringe pump.

For programing the Arduino controller and the touch screen follow the steps 13 to 15.

13. Connect the controller (Arduino Nano) to a computer and upload the provided code in 3 (design files) using Arduino IDE. You may need to install Nextion library (Nextion- master) from GitHub.
14. Connect the touch screen (Nextion Display 2.4″) to the computer using USB-to-TTL adapter and upload the provided code in 3 (design files) using Nextion Editor.
15. As explained in 2.2.3 the user enters the amount of volume in μL and the flow rate in μL/min to be inserted and the flow rate. Using these two inputs along with the rated steps per revolution for the used stepper motor (in this case 30,000 steps / revolution) the duration of each stepper motor pulse is calculated and generated. For example:

V = 200μL

Q=20μL/min

$$N_{\mathrm{rev}}=30,000steps/rev$$

D =8mmwhere V is the volume, Q is the flow rate, $N_{\mathrm{rev}}$ is the number of steps per revolution, D the travel distance per revolution. If the syringe used is 3 ml then every 1.5 mm travelled distance a 100 μL is injected. Hence, to inject 200μL a 3 mm must be crossed. The number of steps needed (N) to inject the wanted volume is:

$$N=\frac{3mm}{D}*N_{\mathrm{rev}}=\frac{3mm}{8mm/rev}*30000steps/rev\approx 11250steps$$

Hence the pulse duration is:

$$P=\frac{V/Q}{N}=\frac{\frac{200\mu L}{20\mu L/min}}{11250steps}*60s=53.3ms$$

The previous calculations are already implemented and calculated by the controller, the user has to insert the volume and flow rate only. The steps per revolution, travel distance per revolution and the size of the syringe pump data are already written inside the code which can edited easily.

### Acrylic cabinet and collector

**Fig. 17:**
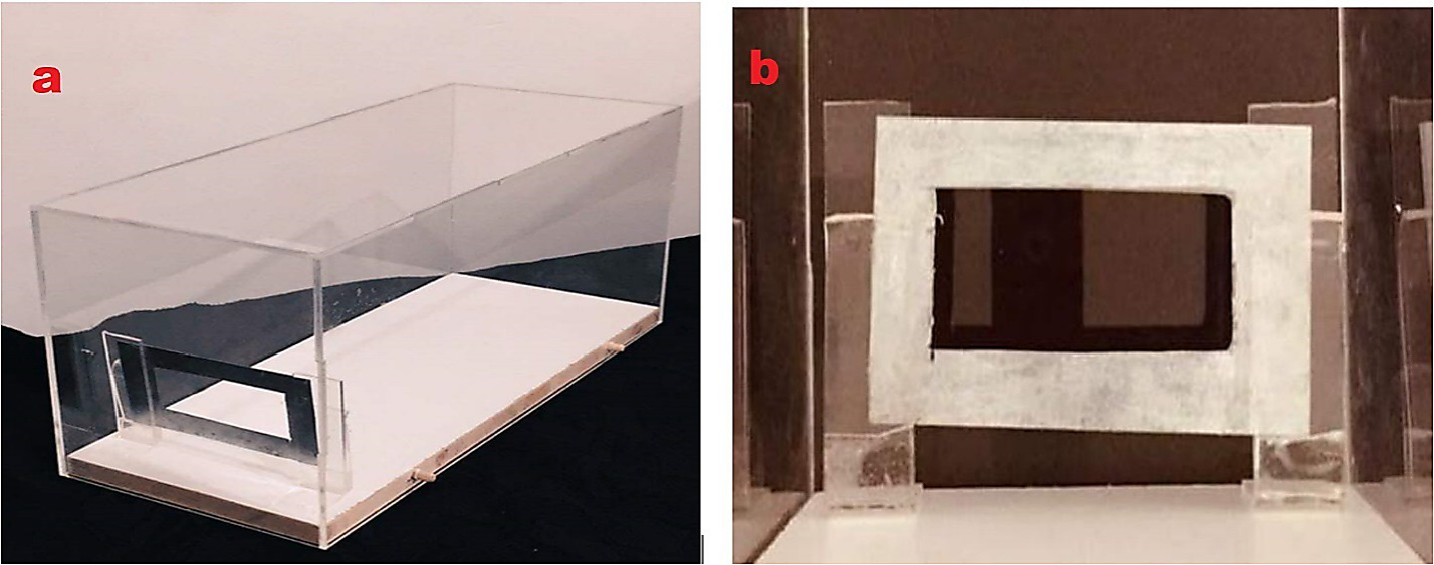
(a) Acrylic cabinet, (b) Collector.

The Acrylic cabinet and collector are the simplest parts to be built in this project. Cabinet and collector are shown in Fig. 17. The current acrylic cabinet and the collectors were built and constructed in university workshop.

## Operation instructions

The operating Instructions for the syringe pump can be divided into three parts. In the first part, the preparation of the polymer and the handling of the syringe is explained. The procedure of setting up the HVPS and its connection is listed in the second part. Finally, the safety aspect and the initiating process is described. After carefully testing each part of the system separately during the building procedure, the following steps should be followed to have nanofiber fabrication:

### Handling of the syringe pump

1. First of all, 2% poly-l,d-lactic acid (PLA) solution was prepared by dissolving PLA granules in a mixture of chloroform plus N,N-Dimethylformamide solvents in a (7:3) ratio. The resulting 0.5 ml PLA solution was loaded to syringe.
2. Let the collector at 15 cm distance from the needle.
3. Turn on the syringe pump, the display will start with the introduction page, after five seconds the display will go to the main page automatically.
4. For example, set the: volume (200 µL), flow rate (025 µL/ minute), and the delay time (90 s). Press the ***return*** button at each page to return to the main page Fig. 11(c), (d), and (e).

### The use of the power supply

1. Connect the autotransformer to the HVPS as shown in Fig. 18(a).
2. Connect the “banana-to-crocodile” wire; the banana side to the power supply. The positive crocodile (red color) must be connected to the needle, and the negative (black color) to the collector. See Fig. 18(b).

**Fig. 18:**
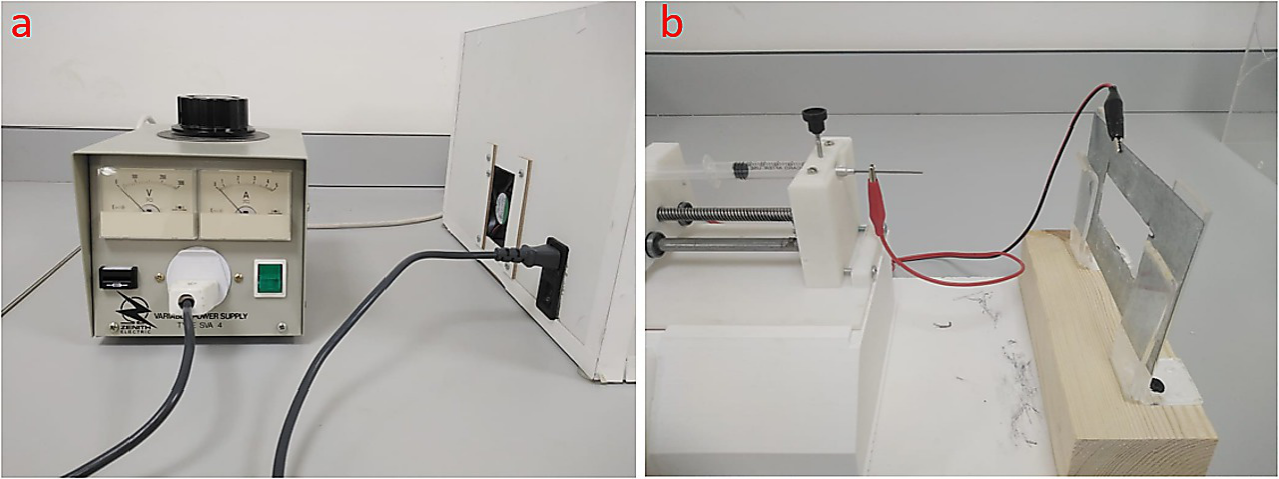
(a) autotransformer connection with HVPS, (b) banana cable connection with needle and collector.
3. Turn on the autotransformer and the HVPS. Make sure that the autotransformer is at zero position (zero volt) before turning it on.

### The safety aspects

1. Cover the syringe pump and the collector by the acrylic box.
2. Increase the voltage using the autotransformer gradually and monitor the voltage at the LCD. Stop whenever you reach the required voltage (±8 kV).
3. Press ***start*** button. The display will go to the monitoring page as shown in Fig. 11(f). You can stop the system at any time or press return to go to the main page.
4. After the time delay, the system will start pumping the polymer and the nanofiber will be fabricated.

Never turn on the HVPS before you cover all the high voltage parts (syringe pump needle & the collector. Set the time delay to be enough to do all steps before start pumping the polymer. It is preferable to have an autotransformer with current monitoring. After the process is completed, reduce the voltage gradually then turn off the power supply and the autotransformer before you remove the acrylic cover. The operation process can be viewed with the supplied demonstration video (https://doi.org/10.17605/OSF.IO/5YGWE).

## Validation and characterization

**Fig. 19:**
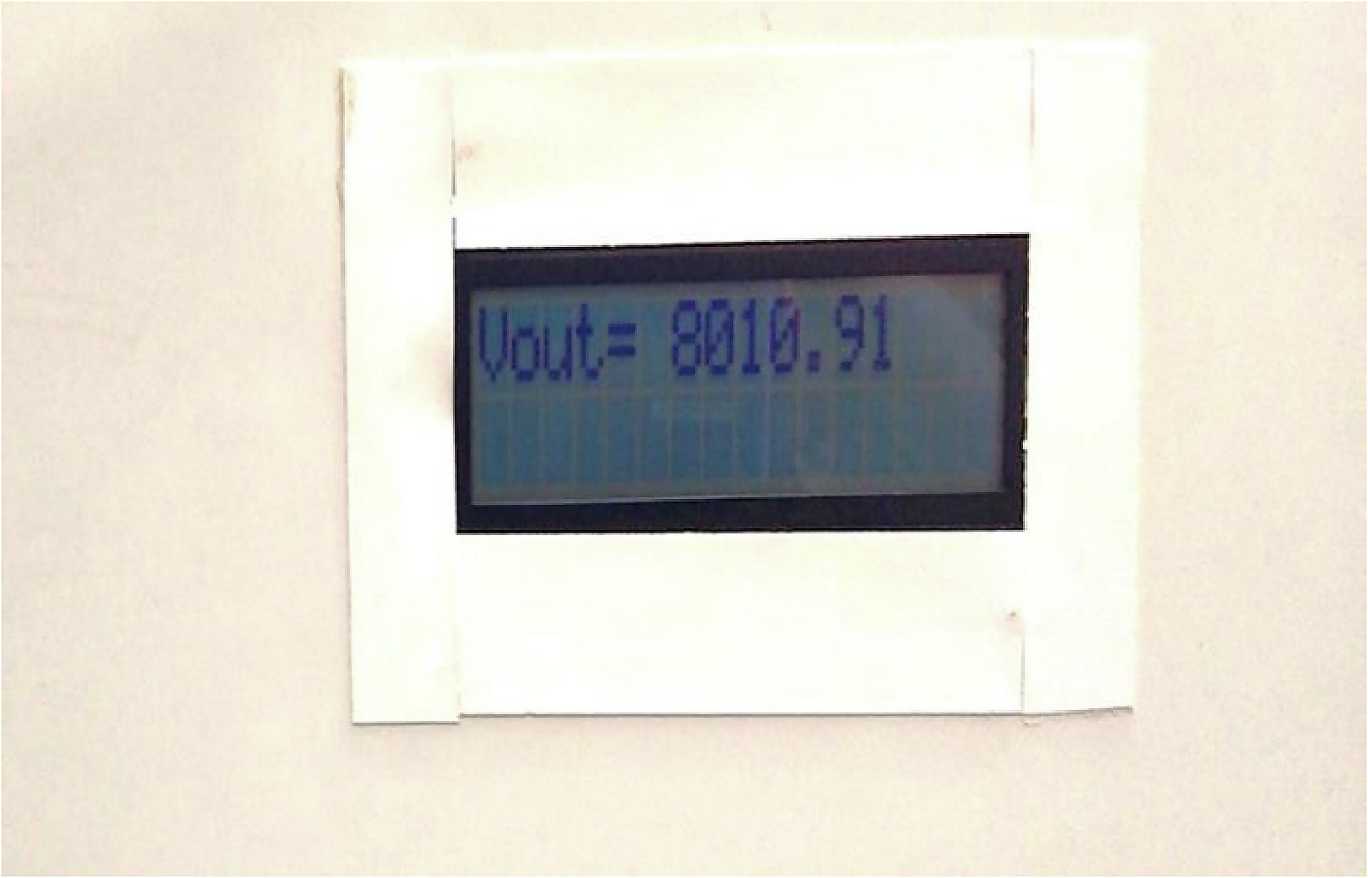
HVPS reading about 8 kV.

The method of electrospinning is used to manufacture nanofibre mesh. The laboratory protocol [9] was modified for nanofibre mesh production. 2% PLA solution was produced by dissolving the PLA material into a mixture of chloroforms and N, N-dimethylformamide (nanofibers solvents in a ratio of 7:3). The resultant 0.5 ml PLA solution was applied by a lab-designed syringe pump through an 18G needle connected to the positive tip at a flow rate of 0.025 ml/minute. The distance from the positive electrode to the collector was 15 cm. Our lab-built power supply with ≃±8 kV power was connected with the handmade collector and tip of needle. For fabricated nanofibre meshes collecting a rectangular handheld collector was built from a conductive material frame connected to the negative electrode. The HVPS is intended to be used in the electrospinning device, and this power is cost-effective in researching and studying the electrospinning machine. The output voltage of up to 8 kV is shown in Fig. 19

**Fig. 20:**
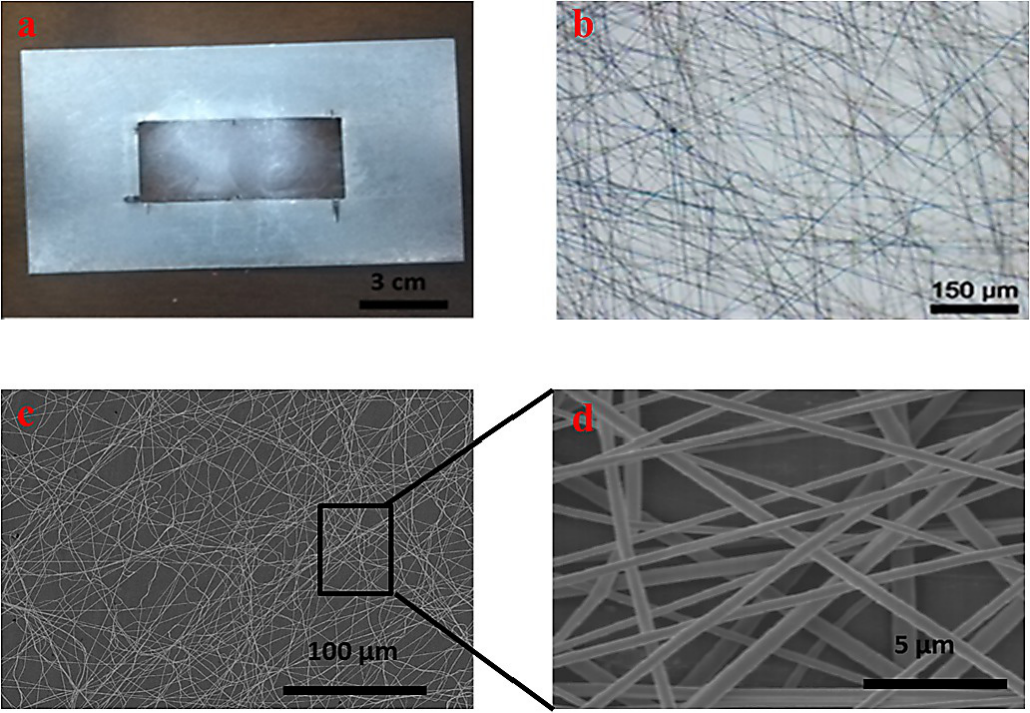
a) Nanofiber’s mesh deposited on collector. b) Bright field image for nanofibers mesh 150 µm scale bar. c) SEM image of fabricated electrospun mesh. Scale bar 100 µm d) SEM image of fabricated electrospun mesh. Scale bar 5 µm.

The scanning electron microscopy (SEM) is used to study the structure, morphology, and diameter of nanofibers. The nanofibers' diameter range was 328 nm to 718 nm determined by analysis of the images by Image J software. At least 15 nanofibers are selected for the calculation, by and the average diameter is 523 nm which is acceptable comparing with previous study [14] which succeed to fabricate nanofibers with 450 nm. Fig. 20 demonstrated nanofibers structure in different magnifications and microscopes. (i.e. naked eyes, bright field, and SEM microscopes) to clarify the fabricated nanofibers' diameter.

## Human and animal rights

No ethical approval needs.

## Declaration of Competing Interest

The authors declare that they have no known competing financial interests or personal relationships that could have appeared to influence the work reported in this paper.
